# Generation of short-term follow-up chest CT images using a latent diffusion model in COVID-19

**DOI:** 10.1007/s11604-024-01699-w

**Published:** 2024-11-25

**Authors:** Naoko Kawata, Yuma Iwao, Yukiko Matsuura, Takashi Higashide, Takayuki Okamoto, Yuki Sekiguchi, Masaru Nagayoshi, Yasuo Takiguchi, Takuji Suzuki, Hideaki Haneishi

**Affiliations:** 1 Department of Respirology, Graduate School of Medicine, Chiba University, 1-8-1, Inohana, Chuo-Ku, Chiba-Shi, Chiba, 260-8677 Japan; 2 Graduate School of Science and Engineering, Chiba University, Chiba, 263-8522 Japan; 3 Center for Frontier Medical Engineering, Chiba University, 1-33, Yayoi-Cho, Inage-Ku, Chiba-Shi, Chiba, 263-8522 Japan; 4 Institute for Quantum Medical Science, National Institutes for Quantum Science and Technology, 4-9-1, Anagawa, Inage-Ku, Chiba-Shi, Chiba, 263-8555 Japan; 5 Department of Respiratory Medicine, Chiba Aoba Municipal Hospital, 1273-2 Aoba-Cho, Chuo-Ku, Chiba-Shi, Chiba, 260-0852 Japan; 6 Department of Radiology, Chiba University Hospital, 1-8-1, Inohana, Chuo-Ku, Chiba-Shi, Chiba, 260-8677 Japan; 7 Department of Radiology, Japanese Red Cross Narita Hospital, 90-1, Iida-Cho, Narita-Shi, Chiba, 286-8523 Japan

**Keywords:** COVID-19, Latent diffusion model, Chest CT images, Prognostic image generation, Deep learning

## Abstract

**Purpose:**

Despite a global decrease in the number of COVID-19 patients, early prediction of the clinical course for optimal patient care remains challenging. Recently, the usefulness of image generation for medical images has been investigated. This study aimed to generate short-term follow-up chest CT images using a latent diffusion model in patients with COVID-19.

**Materials and methods:**

We retrospectively enrolled 505 patients with COVID-19 for whom the clinical parameters (patient background, clinical symptoms, and blood test results) upon admission were available and chest CT imaging was performed. Subject datasets (*n* = 505) were allocated for training (*n* = 403), and the remaining (*n* = 102) were reserved for evaluation. The image underwent variational autoencoder (VAE) encoding, resulting in latent vectors. The information consisting of initial clinical parameters and radiomic features were formatted as a table data encoder. Initial and follow-up latent vectors and the initial table data encoders were utilized for training the diffusion model. The evaluation data were used to generate prognostic images. Then, similarity of the prognostic images (generated images) and the follow-up images (real images) was evaluated by zero-mean normalized cross-correlation (ZNCC), peak signal-to-noise ratio (PSNR), and structural similarity (SSIM). Visual assessment was also performed using a numerical rating scale.

**Results:**

Prognostic chest CT images were generated using the diffusion model. Image similarity showed reasonable values of 0.973 ± 0.028 for the ZNCC, 24.48 ± 3.46 for the PSNR, and 0.844 ± 0.075 for the SSIM. Visual evaluation of the images by two pulmonologists and one radiologist yielded a reasonable mean score.

**Conclusions:**

The similarity and validity of generated predictive images for the course of COVID-19-associated pneumonia using a diffusion model were reasonable. The generation of prognostic images may suggest potential utility for early prediction of the clinical course in COVID-19-associated pneumonia and other respiratory diseases.

**Supplementary Information:**

The online version contains supplementary material available at 10.1007/s11604-024-01699-w.

## Introduction

To date, the coronavirus disease 2019 (COVID-19) pandemic has caused > 770 million infections and 6.9 million deaths worldwide [1]. The World Health Organization (WHO) removed a Public Health Emergency of International Concern (PHEIC) status for COVID-19 in May 2023, while warning that new strains could pose a threat. Despite the return to normalcy, early prediction of patients’ clinical trajectories remains unresolved, impacting appropriate patient care and healthcare resource allocation [2].

Deep learning (DL) methods have gained significant traction in medicine [3, 4]. DL-based analysis of chest images has enabled prediction of COVID-19 disease severity and survival, with many reports showing high diagnostic accuracy [5]. Scoring DL systems trained with clinical data to determine COVID-19 patient status also predicted critical illness [6]. However, DL models combining different types of information remain under-investigated [7]. Furthermore, since the prediction results have been limited to scoring and classification, it would be more useful if both types of information could be utilized to generate short-term follow-up images, i.e., prognostic images.

Generative artificial intelligence (AI) has been developed for many applications globally [8]. For image generation (synthesis), AI can generate new and realistic images from existing datasets. Notably, image generative AI such as a generative adversarial network (GAN) [8] and a variational autoencoder (VAE) [9] were applied to medical imaging [10]. Initially, medical image generation primarily focused on noise reduction and data augmentation. Currently, it is anticipated to evolve into a potent tool for diagnostic imaging, enhancing clinical performance. Studies of chest image generation, employing GANs, have accomplished tasks such as generating pulmonary nodules [11] and diagnosing COVID-19-associated pneumonia in chest CT images [12].

The original concept of diffusion models (DMs) was published in 2015 [13] and the methods have been applied to many fields [14, 15]. DMs can generate a variety of images by encoding textual information along with image information [16]. Better performance was found in histopathology and neurology [17, 18]. In addition, the generation of chest CT images using open datasets has been reported [19].

During the pandemic, reports of image generation using chest CT or X-rays were predominantly performed for data augmentation, lung segmentation and diagnosis [20]. However, prognostic image generation for predicting disease progression remains unexplored. In patients recovering from COVID-19, lung abnormalities on chest CT scans have been reported to peak approximately 10 days after the onset [21]. This study aimed to develop a novel approach integrating clinical data, radiological features, and chest CT images to generate short-term follow-up images. In addition to predicting disease severity, prognostic images can offer more comprehensive information on various morphologic features as a basis for prediction and may contribute to treatment recommendation and decision-making in clinical practice. This tool could potentially help both patients and professionals facing challenges related to COVID-19 and may also be useful in the management of other respiratory diseases.

## Methods

### Study subjects

**Fig. 1:**
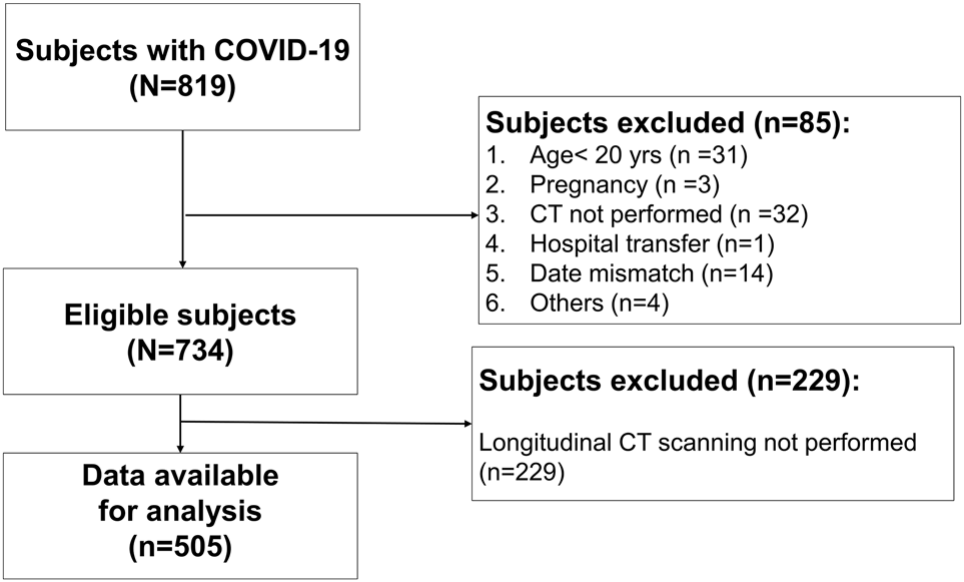
Flowchart of the study subjects. Notes: Of the 819 participants, 85 were excluded due to the following reasons: age < 20 years (*n* = 31), pregnancy (*n* = 3), no CT scans (*n* = 32), hospital transfer (*n* = 1), date mismatch (*n* = 14) and others (*n* = 4). Another 229 patients were excluded because they did not undergo follow-up CT imaging

Initially, 819 consecutive COVID-19 patients admitted and treated at Chiba Aoba Municipal Hospital from February 2020 to September 2021 were enrolled. The inclusion criteria for this retrospective study were patients diagnosed with COVID-19, confirmed by a positive PCR test result and requiring treatment and hospitalization. An initial CT scan was conducted upon admission for most patients, with subsequent follow-up scans to monitor pneumonia progression, including exacerbations. We excluded subjects under 20 years of age (*n* = 31), pregnant subjects (*n* = 3), subjects who did not undergo CT scanning (*n* = 32), a transfer case (*n* = 1), subjects with date mismatches (*n* = 14), other cases with insufficient data for which semi-automatic image preprocessing was required for part of the images (*n* = 4), and subjects who did not undergo follow-up CT scanning (*n* = 229); thus, 505 patients were ultimately enrolled (Fig. 1).

Most participants in the present study had also been analyzed in a previous study with different research objectives [22].

We prepared this article in accordance with the Checklist for Artificial Intelligence in Medical Imaging [23]. The study was conducted in accordance with the principles of the Declaration of Helsinki and was approved by the Institutional Review Boards of Chiba University (No. 4074) and Chiba Aoba Municipal Hospital (No. 20200301). The requirement for written informed consent was waived due to the characteristics of the retrospective study. To avoid any potential breach of patient confidentiality, the data were deidentified and had no link to researchers.

### Clinical information

Data were gathered through patient chart reviews upon admission, encompassing patient background, clinical symptoms, and blood test results. This information was collected within 24 h of the initial visit or admission, comprising 19 background items, 9 clinical symptoms (see Supplementary Table 1 online), and 34 blood test parameters (Supplementary Table 2 online), totaling 62 items. Each patient dataset was normalized and any missing values were filled in using the mode method.

### Chest CT imaging and registration

The initial CT scans utilized 128-row CT scanners (SOMATOM Definition AS, Siemens, Erlangen, Germany) with settings including 120 kV, automatic exposure control, and a gantry rotation time of 0.5 s. Images were reconstructed using soft (I40f) and sharp reconstruction kernel (B70f) at a 3-mm slice thickness. Follow-up CT scans employed 64-row CT scanners (Aquilion CXL, Canon Medical Systems, Otawara, Tochigi, Japan) with settings including 120 kV, automatic exposure control, and a gantry rotation time of 0.5 s. Soft (FC13) and sharp (FC53) kernels were used for image reconstruction at a 3-mm slice thickness. No contrast medium was used. Soft kernels were utilized for model construction, while sharp kernels were used for image data verification and clinical practice, including diagnosis and management. To address scanner-related variances and the effects of non-rigid registration, a Gaussian filter (σ = 1.0) was applied for preprocessing. Images were cropped to encompass the lung fields and resized to 256 × 256 × 128 voxels in line with our operating environment (NVIDIA RTX3090). An affine transformation followed by non-rigid deformation via VoxelMorph [24] facilitated initial alignment and subsequent processing of follow-up CT images.

### Image feature extraction

Image feature extraction utilized PyRadiomics, an open-source package endorsed for standardized radiomics analysis workflow [25, 26]. Radiomics features of clipped lung lesions were extracted, yielding 55 features per lung slice, as detailed in Supplementary Table 3 (online), excluding the uppermost and bottommost lung sections.

### Datasets

Out of the 505 subject datasets, 403 were randomly allocated for training, while the remaining 102 were designated for evaluation in a 4:1 ratio. The total image dataset count was 22,098, with 17,632 used for training and 4466 for validation.

### Adaptation to the diffusion model and image generation

**Fig. 2:**
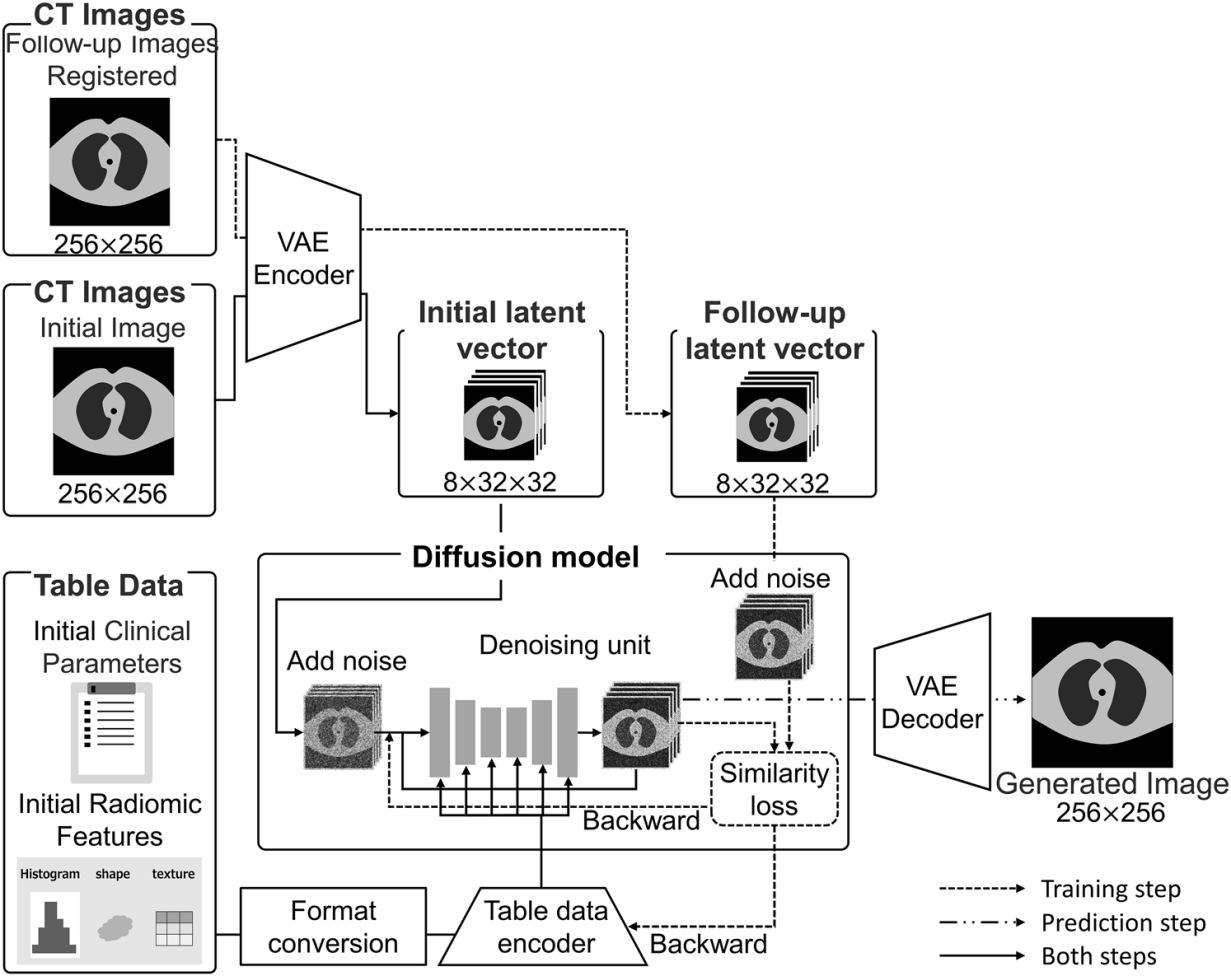
Development of the proposed image generation model and proposed network architecture. Notes: This study introduces a novel deep learning algorithm grounded in the diffusion model (DM) to produce prognostic chest CT images for prediction of COVID-19-associated pneumonia trajectory. In the prediction phase, image generation is facilitated using an evaluation dataset comprising initial CT scans and corresponding tabular data

In this study, we proposed a deep learning algorithm based on DM [16] for prognostic chest CT image generation to predict COVID-19-associated pneumonia (Fig. 2), which incorporated a Connecting Text and Images (CLIP) network [14].

Image data training was performed as follows: initial and follow-up CT image were converted to 256 × 256 × 3 channel images to suit the VAE for color images. The images underwent VAE encoding, resulting in 8 × 32 × 32 latent vectors (solid and dotted lines in Fig. 2). The initial latent vector was input to the diffusion model and underwent noise injection and denoising using U-net [27]. The follow-up latent vector underwent similar processing, and the similarity loss was calculated and backward projected.

**Fig. 3:**
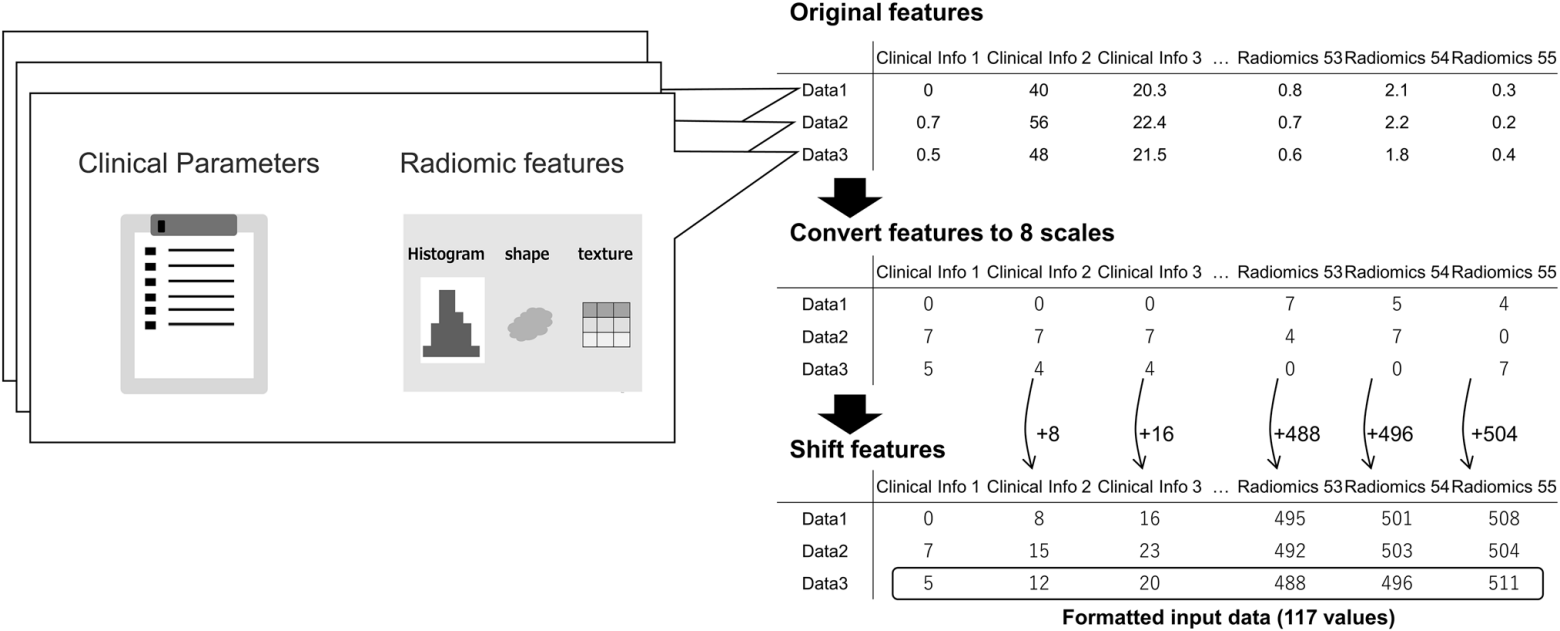
The proposed table data encoder. Notes: The total number of clinical parameters and radiomic features was 117. Each item was normalized and the items were converted into eight scales. We formatted the table data as a table data encoder. The number of words in the prompt was set to 117. The values of each item were sequentially increased by 8, so that each item had a unique value

The training from the table data was performed as follows (Fig. 3). For the datasets, a total of 62 clinical parameters were utilized. In addition, 55 imaging features were computed. A total of 117 table data items were normalized, and the table data were formatted as input for the encoder, with the prompt items set at 117. To ensure that each item had a unique value, the value of each item was sequentially increased by 8. The table data encoder was placed in each layer of the U-net within the model and trained in a closed environment.

The noise scheduler was set to 60 times. The mean squared error (MSE) served as the loss function. During the prediction phase, images were generated using an evaluation dataset comprised of the initial CT image and table data (solid and dashed lines in Fig. 2).

The images were generated from the entire lung, excluding the uppermost and bottommost parts.

### Evaluation of whole image similarity

To assess the image similarities between the generated and the original follow-up (real) images, we computed the zero-mean normalized cross-correlation (ZNCC), peak signal-to-noise ratio (PSNR) and structural similarity (SSIM).

### Visual evaluation of the generative images

Visual assessments were conducted independently by two pulmonologists (NK experience > 20 years, YM experience > 15 years) and one radiologist (KH experience > 20 years) on the 102 cases generated. The evaluation focused on three specific anatomical levels (images): the lower pulmonary vein inlet as representative of the lower lung field, the tracheal branch as representative of the middle-lung field, and the upper end of the arch as representative of the upper lung field. Three types of evaluation criteria were used. First, the shape consistency was assessed across three cross-sectional areas commonly used in clinical image evaluations, including the lower pulmonary vein inlet, tracheal branch, and upper end of the arch. The specific criteria included consistency in (a) the pulmonary vessel direction and branching (b) the bronchial position within the lung field between the follow-up and generated prognostic CT images. Congruence levels were rated as less than 20% (score = 0), 20% to 50% (score = 1), 50% to 80% (score = 2), and 80% or higher (score = 3). Second, the extent of the lesions indicative of COVID-19-associated pneumonia was evaluated for significant differences between the actual follow-up CT and the generated prognostic CT images within the same cross-sections. Pneumonia severity was rated as no pneumonia (score = 0), less than 25% (score = 1), 25% to 50% (score = 2), 50% to 75% (score = 3), and 75% or higher (score = 4). Statistical differences between each of these items were subsequently calculated. Lastly, over- and/or underworking in the generated images was classified as follows [28]: obvious over- and/or underworking (score = 1), some over- and/or underworking (score = 2), a little over- and/or underworking (score = 3); and no over- and/or underworking (score = 4). Then, the mean of the scores of each of the three observers was calculated. Clinical information was not available to the expert readers.

### Statistical analysis

The results were expressed as a mean ± standard deviation (± SD), while categorical data were shown as a number (%). Statistical analyses utilized JMP Pro version 17.0 software (SAS Institute, Cary, NC, USA). Comparisons between training and test datasets for the clinical information used a Mann–Whitney *U* test for the continuous variables and a Chi-square test or Fisher exact test for the categorical variables, after confirming parametricity. In addition, differences in values between the follow-up CT and generated prognostic CT images were analyzed. Two of the authors (N.K. and Y.I.) performed all statistical analyses and the significance level was set at *p* < 0.05.

## Results

### Main demographics of the study participants

The demographic characteristics of the participants are summarized (Table 1). The average patient age was 55 years, with males predominant. The mean time from symptom onset to initial CT was 5.8 days, and initial CT to follow-up CT was 9.6 days. The chief symptom upon admission was fever in 91% of the patients, cough in 58%, dyspnea in 42%, fatigue in 52%, and dysgeusia or dysosmia in 22%. During hospitalization, 210 patients (42%) received oxygenation, with 24 (5%) on oxygenation at admission. High-flow nasal cannula (HFNC) therapy was administered to 68 patients (13%), while 18 patients (4%) required intubation. Two patients needed extracorporeal membrane oxygenation (ECMO). Thirteen patients did not survive. Except for alcohol consumption and symptom-free percentage, no significant differences were observed between the training and test datasets.

**Table 1:** Main demographics of the study subjects (*n* = 505)

	Total subjects (*n* = 505) *n* (%, percentage)	Training dataset (*n* = 403)	Test dataset (*n* = 102)
Age	55 (20–97)	55(20–97)	52(21–86)
Gender (male)	330 (65%)	263(65%)	67(66%)
BMI	25.2 ± 4.4	25.3 ± 4.5	24.9 ± 4.3
Current smoker	149 (30%)	118(29%)	31(30%)
Pack-years	12.7 ± 22.0	13.1 ± 22.2	11.1 ± 21.1
Alcohol consumption	277 (55%)	231(57%)	46(45%)*
Symptom onset to initial CT (days)	5.8 ± 3.2	5.7 ± 3.2	6.2 ± 3.3
Range (days)	(0–19)	(0–17)	(0–19)
Initial CT to follow-up CT (days)	9.6 ± 7.2	9.7 ± 7.4	9.2 ± 6.7
Range (days)	(2–58)	(2–58)	(2–44)
*Co-morbidities*			
Hypertension	139 (28%)	116(29%)	23(23%)
Diabetes mellitus	81 (16%)	62(15%)	19(19%)
Dyslipidemia	76 (15%)	62(15%)	14(14%)
Coronary disease	14 (3%)	12(3%)	2(2%)
Bronchial asthma	27 (5%)	24(6%)	3(3%)
COPD	4 (0.8%)	3(0.7%)	1(1%)
*Symptom*			
Fever	458 (91%)	366(91%)	92(90%)
Cough	288 (58%)	232(58%)	56(55%)
Dyspnea	214 (42%)	175(43%)	39(38%)
Fatigue	264 (52%)	207(51%)	57(56%)
Sore throat	136 (27%)	106(26%)	30(29%)
Diarrhea	60 (12%)	50(12%)	10(10%)
Nausea/vomiting	41 (8%)	29(7%)	12(12%)
Dysgeusia/dysosmia	111 (22%)	88(22%)	23(23%)
None	4 (0.8%)	1(0.2%)	3(3%)**
*Outcome*			
Oxygen supplementation	210 (42%)	170(42%)	40(39%)
Oxygen supplementation at time of hospital admission	24 (5%)	21(5%)	3(3%)
High-flow nasal oxygen	68 (13%)	57(14%)	11(11%)
Intubation	18 (4%)	14(3%)	4(4%)
ECMO	2 (0.4%)	2(0.5%)	0(%)
Survival/death	492 (97%)/ 13 (3%)	392(97%)/11 (3%)	100(98%)/2 (2%)

Note: Data are expressed as the mean ± standard deviation

The difference between the training dataset and the test dataset

*p < 0.05; **p < 0.01;

*BMI* body mass index, *COPD* chronic obstructive pulmonary disease, *ECMO* extracorporeal membrane oxygenation, *NA* not assessed

At admission, the aspartate aminotransferase (AST), alanine aminotransferase (ALT), lactate dehydrogenase (LDH), γ-glutamyltransferase (γ-GTP), C-reactive protein (CRP), blood glucose, and D-dimer levels were slightly elevated compared to normal ranges (Table 2). However, no significant differences were observed between the training and test datasets for these levels.

**Table 2:** Major blood test findings of the study subjects (*n* = 505)

Laboratory indices	Total subjects (*n* = 505)	Training dataset (*n* = 403)	Test dataset (*n* = 102)
TP (g/dL)	7.0 ± 0.6	7.1 ± 0.6	7.0 ± 0.6
ALB (g/dL)	3.9 ± 0.5	3.9 ± 0.5	3.9 ± 0.6
AG ratio	1.3 ± 0.3	1.3 ± 0.2	1.3 ± 0.2
AST (IU/L)	43.9 ± 34.7	45.3 ± 36.1	38.3 ± 27.7
ALT (IU/L)	40.2 ± 38.9	40.8 ± 40.0	37.9 ± 34.4
LDH (U/dL)	322.9 ± 151.7	329.9 ± 160.3	295.3 ± 107.7
T-Bil (mg/dL)	0.66 ± 0.3	0.68 ± 0.4	0.60 ± 0.2
γ-GTP (IU/L)	79.6 ± 118.7	81.6 ± 127.3	71.8 ± 75.6
BUN (mg/dL)	15.0 ± 7.6	15.1 ± 7.5	14.7 ± 8.0
Cre (mg/dL)	0.86 ± 0.3	0.86 ± 0.3	0.87 ± 0.5
UA (mg/dL)	4.6 ± 1.6	4.6 ± 1.7	4.5 ± 1.4
eGFR (mL/min/1.73 m^2^)	73.4 ± 18.6	72.9 ± 18.2	74.9 ± 20.0
Na (mEq/L)	137.1 ± 3.7	137.0 ± 3.8	137.2 ± 3.4
K (mEq/L)	4.0 ± 0.4	4.0 ± 0.4	4.0 ± 0.4
Cl (mEq/L)	100.2 ± 4.2	100.2 ± 4.3	100.5 ± 3.8
CPK (U/L)	167.1 ± 333.8	181.3 ± 369.7	111.4 ± 95.7
CRP (mg/dL)	4.8 ± 4.8	4.9 ± 4.9	4.2 ± 4.4
GLU (mg/dL)	130.4 ± 51.5	128.1 ± 45.8	139.8 ± 69.8
WBC count (/μL)	5411 ± 2217	5461 ± 2236	5211 ± 2140
RBC count (× 10^4^/μL)	479.9 ± 56.6	479.8 ± 56.2	480.1 ± 58.3
HGB (g/dL)	14.5 ± 1.8	14.6 ± 1.7	14.5 ± 1.8
Hct (%)	42.2 ± 4.7	42.2 ± 4.6	42.0 ± 4.8
MCV (fL)	88.1 ± 4.9	88.2 ± 5.1	87.7 ± 4.0
MCH (pg)	30.4 ± 2.0	30.4 ± 2.2	30.2 ± 1.5
MCHC (%)	34.5 ± 1.2	34.4 ± 1.2	34.5 ± 1.1
PLT (× 10^4^/μL)	19.8 ± 7.0	19.8 ± 6.9	19.5 ± 7.1
Baso (%)	0.36 ± 0.36	0.37 ± 0.38	0.32 ± 0.25
Eosino (%)	0.73 ± 1.33	0.72 ± 1.34	0.78 ± 1.32
Neutro (%)	70.0 ± 11.5	70.3 ± 11.6	68.9 ± 11.0
Lympho (%)	21.7 ± 9.7	21.5 ± 9.8	22.5 ± 9.4
Mono (%)	7.1 ± 3.2	7.2 ± 3.2	6.9 ± 2.9
Neutro count (/μL)	3801 ± 1905	3821 ± 1888	3720 ± 1980
PNI	39.3 ± 5.8	39.3 ± 5.5	39.0 ± 6.9
D-dimer (ug/mL)	1.9 ± 10.9	1.4 ± 3.6	2.2 ± 9.9

Note: Data are expressed as the mean ± standard deviation. No items had a significant difference between the training dataset and the test dataset

*TP* total protein, *ALB* albumin, *AG ratio* albumin:globulin ratio, *AST* aspartate aminotransferase, *ALT* alanine aminotransferase, *LDH* lactate dehydrogenase, *T-Bil* total bilirubin, *γ-GTP* γ-glutamyltransferase, *BUN* blood urea nitrogen, *Cre* creatinine, *UA* uric acid, *eGFR* estimated glomerular filtration rate, *Na* sodium, *K* potassium, *Cl* chloride ion, *CPK* creatine phosphokinase, *CRP* C-reactive protein, *GLU* glucose, *WBC* white blood cell, *RBC* red blood cell, *HGB* hemoglobin, *Hct* hematocrit, *MCV* mean corpuscular volume, *MCH* mean corpuscular hemoglobin,

*MCHC* mean corpuscular hemoglobin concentration, *PLT* platelet, *Baso* basophil: Eosino, eosinophil, *Neutro* neutrophil, *Lympho* lymphocyte, *Mono* monocyte, *PNI* prognostic nutritional index, *NA* not assessed

### Examples of generated prognostic images

**Fig. 4:**
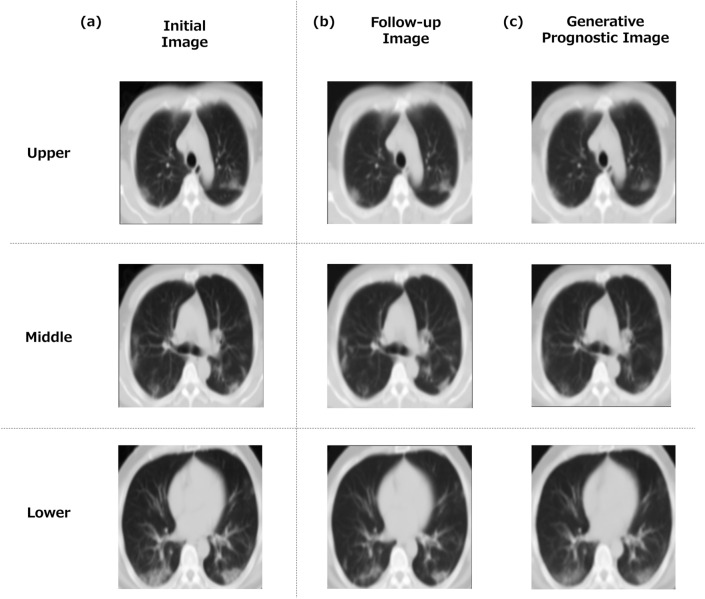
An example of a generated prognostic CT image (moderate case). Notes: A moderate case of a 38-year-old man requiring oxygenation. The initial CT was performed on day 6 after the onset of symptoms, and the follow-up CT was performed on day 11. Similarities between the generated prognostic image and the follow-up image are as follows: in the upper lung field, 0.988 in ZNCC, 26.70 in PSNR, 0.902 in SSIM, in the mid-lung field, 0.991 in ZNCC, 27.74 in PSNR, 0.899 in SSIM, and in the lower lung field, 0.984 in ZNCC, 24.99 in PSNR, 0.849 in SSIM. Overall, 0.988 in ZNCC, 26.48 in PSNR, 0.883 in SSIM. The visual evaluation by the experts was as follows. In the upper lung field, the similarity of lung structures in the generated image was 3.0 out of 3. The degree of pneumonia was 1.0 out of 4 for the follow-up images and 1.0 out of 4 for the generated images. The over/under evaluation of the image was 3.7 out of 4. In the mid-lung field, the similarity of lung structures in the generated image was 3 out of 3. The degree of pneumonia was 1.0 out of 4 for the follow-up image and 1.0 out of 4 for the generated images. The over/under evaluation of the image was 3.3 out of 4. In the lower lung field, the similarity of lung structure in the generated image was 3.0 out of 4. The degree of pneumonia was 1.0 out of 4 for the follow-up image and 1.0 out of 4 for the generated images. The over/under evaluation of the image was 3.7 out of 4. Overall, the similarity of lung structures in the generated images was rated 3.0 out of 3. The pneumonia severity scored 1.0 out of 4 for the follow-up images and 1.0 out of 4 for the generated images. The over/under evaluation of the images was 3.6 out of 4. **a** Initial image, **b** follow-up CT image (real image), **c** generated prognostic CT image (generated image)

Prognostic images generated for both moderate and severe cases are presented. An example of the generated images is shown (Fig. 4) as a moderate case from a 38-year-old man requiring oxygenation. A CT scan was initially performed on day 6 following the onset of symptoms, with a follow-up scan performed on day 11. Three anatomically representative images (upper, middle and lower lung fields) are shown. The similarities of the three images between the generated prognostic image and the follow-up image were quantified as ZNCC = 0.988, PSNR = 26.48, and SSIM = 0.883. Expert visual assessments of the three images were also performed. Overall, the similarity of the lung structures in the generated images was rated 3.0 out of 3. The distribution of pneumonia in the follow-up and generated images both scored 1.0 out of 4. The over/under evaluation of the generated images was rated 3.6 out of 4.

**Fig. 5:**
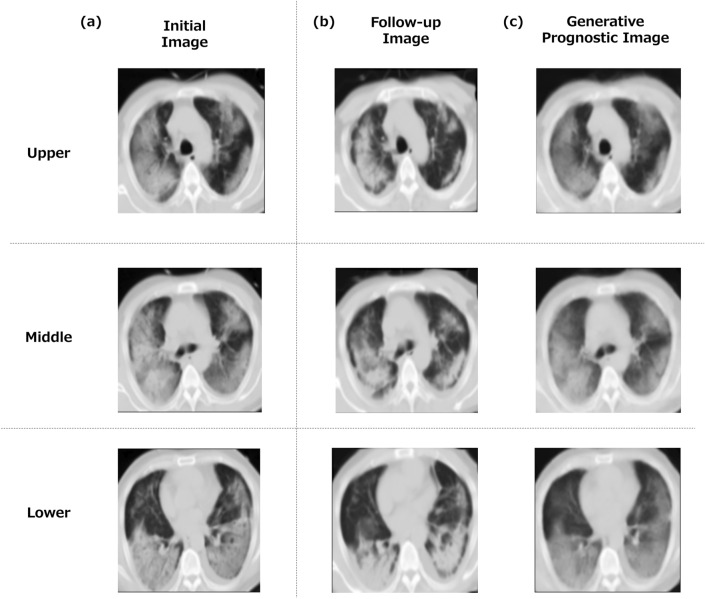
A generated prognostic CT image (severe case). Notes: A critical case of a 56-year-old man who required intensive care including HFNC and intubation. The initial CT was performed on day 3 after the onset of symptoms, and the follow-up CT was performed on day 10. The similarity between the generated prognostic image and the follow-up image were as follows: in the upper lung field, 0.924 in ZNCC, 18.97 in PSNR, 0.666 in SSIM, in the mid-lung field, 0.922 in ZNCC, 20.07 in PSNR, 0.677 in SSIM, and in the lower lung field, 0.913 in ZNCC, 19.61 in PSNR, 0.644 in SSIM. Overall, 0.920 in ZNCC, 19.55 in PSNR, 0.662 in SSIM. The visual evaluation by the experts was as follows. In the upper lung field, the similarity of lung structures in the generated image was 2.7 out of 3. The degree of pneumonia was 3.0 out of 4 for the follow-up image and 3.0 out of 4 for the generated image. The over/under evaluation of the image was 3.0 out of 4. In the middle-lung field, the similarity of lung structures in the generated image was 2.3 out of 3. The degree of pneumonia was 3.0 out of 4 for the follow-up image and 3.0 out of 4 for the generated image. The over/under evaluation of the image was 3.0 out of 4. In the lower lung field, the similarity of lung structure in the generated image was 2.7 out of 4. The degree of pneumonia was 3.0 out of 4 for the follow-up image and 3.0 out of 4 for the generated image. The over/under evaluation of the image was 3.0 out of 4. Overall, the similarity of lung structures in the generated images rated 2.7 out of 3. The pneumonia severity scored 2.8 out of 4 for the follow-up images and 2.9 out of 4 for the generated images. The over/under evaluation of the images was 3.1 out of 4. (a) initial image, (b) follow-up CT image (real image), (c) generated prognostic CT image (generated image)

Another example of the generated images is illustrated in Fig. 5. This was a critical case of a 56-year-old man who required intensive care including HFNC and intubation. A CT scan was initially performed on day 3 following the onset of symptoms, with a follow-up scan performed on day 10. The similarities of the three images between the generated prognostic image and the follow-up image were quantified as ZNCC = 0.920, PSNR = 19.55, and SSIM = 0.662. Expert visual assessments of the three images were also performed. Overall, the similarity of lung structures in the generated images was rated 2.7 out of 3. The pneumonia distribution scored 2.8 out of 4 for the follow-up images and 2.9 out of 4 for the generated images. The over/under evaluation of the images was rated 3.1 out of 4. The ZNCC, PSNR, and SSIM were lower than in the moderate case described above, as were the similarity of the lung structures and the over/under evaluation by expert visual assessments.

### Whole image similarities between the generated prognostic and follow-up images

The whole image similarities between the generated prognostic images and the follow-up images were calculated by the ZNCC, PSNR, and SSIM. For the image similarity, the ZNCC, PSNR, and SSIM showed reasonable values, at 0.973 ± 0.028 for the ZNCC, 24.48 ± 3.46 for the PSNR, and 0.844 ± 0.075 for the SSIM.

### Evaluation of generated images by experts

The visual evaluation of the generated images by two pulmonologists and one radiologist is shown (Table 3). The evaluation of the similarity of the lung structure in the generated images was well considered in the three slices and overall (upper, 2.9 ± 0.2; middle, 2.9 ± 0.3; lower, 2.9 ± 0.2; overall, 2.9 ± 0.2) out of 3. Comparison of the extent of abnormal lesions in the generated prognostic and follow-up images showed a slightly lower degree of abnormal lesions in the prognostic image, but no significant difference between the three slices and overall (upper, 1.0 ± 0.6 versus 1.1 ± 0.6; middle, 0.7 ± 0.7 versus 0.9 ± 0.7; lower, 1.0 ± 0.6 versus 1.1 ± 0.6; overall, 0.8 ± 0.6 versus 0.9 ± 0.6, *p* > 0.05, respectively, for each comparison). The evaluation of over- and under-representation in the generated images was addressed to some extent in the three slices and overall (upper, 2.8 ± 0.7; middle, 3.0 ± 0.7; lower, 2.8 ± 0.7; overall, 3.0 ± 0.7) out of 4.

**Table 3:** Expert evaluation of generated images relative to real images (*n* = 102)

	Upper	Middle	Lower	Total
Similarity of lung structure of generated images relative to follow-up (real) images (0–3)	2.9 ± 0.2	2.9 ± 0.3	2.9 ± 0.2	2.9 ± 0.2
Extent of abnormal lesion in generated images (0–4)	1.0 ± 0.6	0.7 ± 0.7	1.0 ± 0.6	0.8 ± 0.6
Extent of abnormal lesion in follow-up (real) images (0–4)	1.1 ± 0.6	0.9 ± 0.7	1.1 ± 0.6	0.9 ± 0.6
Over/under evaluation of generated images relative to follow-up (real) images (1–4)	2.8 ± 0.7	3.0 ± 0.7	2.8 ± 0.7	3.0 ± 0.7

**Note:** Scores are listed as mean ± standard deviation

The difference between the extent of abnormal lesions in real versus generative images was not significant in each slice, respectively

*Upper* upper lung field, *Middle* middle-lung field, *Lower* lower lung field, *Total* Average of three slices

## Discussion

In this study, we proposed a novel approach employing a DM model to generate prognostic images for patients with COVID-19. The prognostic image similarity was reasonable, along with a reasonable expert score in the visual assessment. The DM-based prognostic image generation utilizing chest CT images and clinical parameters showed the potential to provide clinical utility for COVID-19-associated pneumonia.

PCR testing and chest imaging were both valuable in diagnosis and treatment during the COVID-19 pandemic. Numerous reports highlighted the potential usefulness of AI in the assessment of COVID-19 [29]. DL models combining chest CT images and clinical data have been proposed for severity prediction [30], i.e., classification tasks in DL. However, there are no reports on the generation of clinical prognostic images in COVID-19. Pandemic diseases such as COVID-19 have posed serious challenges, including surges in patient numbers, resource shortages, and healthcare provider exhaustion [29]. We propose that generating prognostic images and integrating them into medical practice would provide a number of morphological features and a basis to enhance clinical diagnostic performance. This would lead to more reliable prognoses, appropriate treatment strategies and presentation of options. Furthermore, predictive factors for long COVID have been reported [31, 32]. It is critical to identify such contributing factors to assess the patients with long COVID as well as survivors. Regarding chest CT findings in long COVID, ground-glass opacities (GGO), fibrotic-like changes, bronchiectasis, interlobular septal thickening, reticular opacities, and infiltrative shadows have been documented [33, 34]. In addition, the generation of predictive images and prognosis prediction for ARDS and respiratory diseases accompanied by acute respiratory failure could be of great value in emergency medicine. Recently, predictive factors and machine learning models for diagnosis and management of ARDS have been reported [35, 36]. Given that predictive follow-up imaging for long COVID and ARDS has not yet been established, we consider this a challenging but clinically significant issue. We aim to investigate this further in the near future.

The generated images demonstrated reasonable similarity to the follow-up images. Compared to prior studies using GANs and DL models [28, 37], the present results were similar or relatively lower. However, they were reasonable given the nature of the task and may indicate valid image quality from the DL-based system. Experts also rated the images as similar and clinically valid upon visual assessment. While a correlation between visual grading and image similarity indices such as PSNR or SSIM has been noted previously [38], it is crucial for experienced clinicians and radiologists to determine the clinical suitability of the generated images. During the pandemic, there were numerous studies on image generation for COVID-19-associated pneumonia using GANs, however, there were only limited evaluations by radiologists or clinical experts, accounting for only 2 out of 57 studies [20]. This lack of evaluation hampers the clinical application of generated medical images. Scores for the over/under evaluation of generated images relative to follow-up images were 3.0 out of 4, indicating a similarity with previous image generation reports [28]. The expert evaluation showed the potential clinical usefulness of the generated prognostic images for COVID-19.

As shown in Figs. 4 and 5 and Table 3, the lung structures on the generated images were generally similar to the real images, and it was also valid in terms of the pneumonia distribution. However, the generated images appear less distinct, with some depicting pneumonia contraction shadows faintly. This is partly attributed to image preprocessing. The follow-up images were obtained using a different imaging device than the initial CT images. We considered it necessary to adjust for the effects of image quality discrepancies resulting from the differences in imaging equipment [39]. In addition, a non-rigid registration process was used to align the follow-up images with the initial images. This registration process involved subpixel interpolation, resulting in a blurring effect in the follow-up images compared to their original quality. Consequently, direct use of the initial and non-rigidly deformed follow-up images for image generation could lead to detrimental differences in sharpness between the two image sets. Therefore, a Gaussian filter was applied to the images to mitigate these effects. Our emphasis was on the way that the lungs and the overall image changed over time, rather than focusing on detailed lung structures. It will be necessary to address blurring of the images in the near future.

This study had several limitations. First, our data were derived from a relatively small number of patients compared to general image generation studies. Our datasets may not have been sufficient to capture the change trends in the deteriorated areas accurately, given the considerable variability in lesion progression among patients. Second, this retrospective study was conducted from the start of the pandemic, and while the CT scanning conditions were consistent, the timing of scans was not precisely standardized across patients, although relative uniformity was maintained during hospitalization. Third, while the study timeframe allowed an estimation of the COVID-19 strain type, strain identification was not feasible. We speculate that the data were primarily acquired up to the epidemic period of the Delta strain, known for its high probability of severe disease. This developed method needs to be validated with Omicron and other emerging strains. Lastly, the inherent limitations of radiation-based imaging modalities are acknowledged. We utilized CT data performed to confirm the clinical course of COVID-19 patients during a never-before-experienced pandemic. Moving forward, considerations for low-dose CT usage and precise imaging intervals are warranted. These preliminary findings need to be confirmed in larger multicenter longitudinal cohorts with adequate ethical considerations.

## Conclusion

The role of generative AI in medical imaging is poised for expansion. Prognostic image generation through DMs may provide potential usefulness in predicting the clinical trajectory of COVID-19 and other respiratory diseases. It will necessitate sustained collaboration between clinicians, radiologists, radiographers and medical image researchers to successfully integrate generative medical images into clinical practice.

## Supplementary Information

Below is the link to the electronic supplementary material.

Supplementary file1 (DOCX 22 KB)

Supplementary file2 (DOCX 21 KB)

Supplementary file3 (DOCX 16 KB)

## Abbreviations

COVID-19: Coronavirus disease 2019
DL: Deep learning
DM: Diffusion model
PSNR: Peak signal-to-noise ratio
SSIM: Structural similarity
ZNCC: Zero mean normalized cross-correlation
