# Supplementary material for: Generation of short-term follow-up chest CT images using a latent diffusion model in COVID-19
Source: Jpn J Radiol. 2024 Nov 25;43(4):622–33. doi: 10.1007/s11604-024-01699-w (PMC11953082; doi:10.1007/s11604-024-01699-w)
Supplement: Supplementary file 1 — Supplementary file1 (DOCX 22 KB) [file 11604_2024_1699_MOESM1_ESM.docx]

**Supplementary Table 1. Breakdown of items used as clinical parameters**

Patient background and symptoms (n = 28)

| **Variables** |  |
| --- | --- |
| Age | Integer value |
| Gender | Categorical, Male, Female |
| Height | Integer value |
| Body weight | Continuous value |
| BMI | Continuous value |
| Current smoker | Categorical: Yes, No |
| Pack-years | Integer value |
| Alcohol consumption | Categorical: Yes, No |
| Symptom onset to CT (days) | Integer value |
| **Co-morbidities** |  |
| Hypertension | Categorical: Yes, No |
| Diabetes mellitus | Categorical: Yes, No |
| Dyslipidemia | Categorical: Yes, No |
| Coronary disease | Categorical: Yes, No |
| Bronchial asthma | Categorical: Yes, No |
| COPD | Categorical: Yes, No |
| Interstitial pneumonia | Categorical: Yes, No |
| Lung cancer | Categorical: Yes, No |
| Pneumothorax | Categorical: Yes, No |
| Other diseases | Categorical: Yes, No |
| **Symptoms** |  |
| Fever ( > 37C) | Categorical: Yes, No |
| Cough | Categorical: Yes, No |
| Dyspnea | Categorical: Yes, No |
| Fatigue | Categorical: Yes, No |
| Sore throat | Categorical: Yes, No |
| Diarrhea | Categorical: Yes, No |
| Nausea/ vomiting | Categorical: Yes, No |
| Dysgeusia/dysosmia | Categorical: Yes, No |
| None | Categorical: Yes, No |

Abbreviations: BMI, body mass index; COPD, chronic obstructive pulmonary disease; ECMO, extracorporeal membrane oxygenation;
