# Supplementary material for: Generation of short-term follow-up chest CT images using a latent diffusion model in COVID-19
Source: Jpn J Radiol. 2024 Nov 25;43(4):622–33. doi: 10.1007/s11604-024-01699-w (PMC11953082; doi:10.1007/s11604-024-01699-w)
Supplement: Supplementary file 2 — Supplementary file2 (DOCX 21 KB) [file 11604_2024_1699_MOESM2_ESM.docx]

**Supplementary Table 2. Breakdown of blood test findings used as clinical parameters**

Blood test findings (n = 34)

| **Laboratory indices** |
| --- |
| TP (g/dL) |
| ALB (g/dL) |
| AG ratio |
| AST (IU/L) |
| ALT (IU/L) |
| LDH (U/dL) |
| T-Bil (mg/dL) |
| γ-GTP (IU/L) |
| BUN (mg/dL) |
| Cre (mg/dL) |
| UA (mg/dL) |
| eGFR (ml/min/1.73 m^2^) |
| Na (mEq/L) |
| K (mEq/L) |
| Cl (mEq/L) |
| CPK (U/L) |
| CRP (mg/dL) |
| GLU (mg/dL) |
| WBC count (/μL) |
| RBC count (×10^4^/μL) |
| HGB (g/dL) |
| Hct (%) |
| MCV (fL) |
| MCH (pg) |
| MCHC (%) |
| PLT (×10^4^/μL) |
| Baso (%) |
| Eosino (%) |
| Neutro (%) |
| Lympho (%) |
| Mono (%) |
| Neutro count (/μL) |
| PNI |
| D-dimer (ug/mL) |

Abbreviations: TP, total protein; ALB, albumin; AG ratio, albumin:globulin ratio; AST, aspartate aminotransferase; ALT, alanine aminotransferase; LDH, lactate dehydrogenase; T-Bil, total bilirubin; γ-GTP, γ-glutamyltransferase; BUN, blood urea nitrogen;

Cre, creatinine; UA, uric acid; eGFR, estimated glomerular filtration rate;

Na, sodium; K, potassium; Cl, chloride ion; CPK, creatine phosphokinase; CRP, C-reactive protein; GLU, glucose; WBC, white blood cell; RBC, red blood cell; HGB, hemoglobin;

Hct, hematocrit; MCV, mean corpuscular volume; MCH, mean corpuscular hemoglobin;

MCHC, mean corpuscular hemoglobin concentration; PLT, platelet; Baso, basophil: Eosino, eosinophil; Neutro, neutrophil; Lympho, lymphocyte; Mono, monocyte; PNI, prognostic nutritional index
