# Supplementary material for: Generation of short-term follow-up chest CT images using a latent diffusion model in COVID-19
Source: Jpn J Radiol. 2024 Nov 25;43(4):622–33. doi: 10.1007/s11604-024-01699-w (PMC11953082; doi:10.1007/s11604-024-01699-w)
Supplement: Supplementary file 3 — Supplementary file3 (DOCX 16 KB) [file 11604_2024_1699_MOESM3_ESM.docx]

**Supplementary Table 3**: Breakdown of Radiomic features (n = 55)

| **Radiomic Features** |
| --- |
| 10Percentile |
| 90Percentile |
| Energy |
| InterquartileRange |
| Kurtosis |
| Maximum |
| MeanAbsoluteDeviation |
| Mean |
| Median |
| Minimum |
| Range |
| RobustMeanAbsoluteDeviation |
| RootMeanSquared |
| Skewness |
| TotalEnergy |
| Variance |
| Elongation |
| MajorAxisLength |
| MaximumDiameter |
| MeshSurface |
| MinorAxisLength |
| Perimeter |
| PerimeterSurfaceRatio |
| PixelSurface |
| Sphericity |
| GrayLevelNonUniformity |
| LongRunEmphasis |
| LongRunHighGrayLevelEmphasis |
| LongRunLowGrayLevelEmphasis |
| RunEntropy |
| RunLengthNonUniformity |
| RunLengthNonUniformityNormalized |
| RunPercentage |
| RunVariance |
| ShortRunEmphasis |
| ShortRunHighGrayLevelEmphasis |
| ShortRunLowGrayLevelEmphasis |
| LargeAreaEmphasis |
| LargeAreaHighGrayLevelEmphasis |
| LargeAreaLowGrayLevelEmphasis |
| SmallAreaEmphasis |
| SmallAreaHighGrayLevelEmphasis |
| SmallAreaLowGrayLevelEmphasis |
| ZonePercentage |
| ZoneVariance |
| DependenceEntropy |
| DependenceNonUniformity |
| DependenceNonUniformityNormalized |
| DependenceVariance |
| LargeDependenceEmphasis |
| LargeDependenceHighGrayLevelEmphasis |
| LargeDependenceLowGrayLevelEmphasis |
| SmallDependenceEmphasis |
| SmallDependenceHighGrayLevelEmphasis |
| SmallDependenceLowGrayLevelEmphasis |
